# Evolution of Carpal Tunnel Syndrome Treatment: A Narrative Review

**DOI:** 10.3390/neurosci7010010

**Published:** 2026-01-12

**Authors:** Đula Đilvesi, Bojan Jelača, Aleksandar Knežević, Željko Živanović, Veljko Pantelić, Jagoš Golubović

**Affiliations:** 1 Faculty of Medicine, University of Novi Sad, Hajduk Veljkova 3, 21000 Novi Sad, Serbia; 2 Department of Neurosurgery, University Clinical Centre of Vojvodina, Hajduk Veljkova 1, 21000 Novi Sad, Serbia; 3 Department for Physical Therapy and Rehabilitation, University Clinical Centre of Vojvodina, Hajduk Veljkova 1, 21000 Novi Sad, Serbia; 4 Department for Neurology, University Clinical Centre of Vojvodina, Hajduk Veljkova 1, 21000 Novi Sad, Serbia

**Keywords:** carpal tunnel syndrome, entrapment neuropathy, wrist splinting, corticosteroid injection, physical therapy, open carpal tunnel release, endoscopic carpal tunnel release, minimally invasive surgery, ultrasound diagnosis, regenerative therapy, neuromobilization, long-term outcomes

## Abstract

Carpal tunnel syndrome (CTS) is the most common peripheral nerve entrapment disorder, with a lifetime prevalence estimated at approximately 10%. This narrative review explores the historical evolution, current management strategies, and emerging trends in CTS diagnosis and treatment. Early recognition of CTS led to the development of conservative interventions, including splinting, corticosteroid injections, and physical therapy, aimed at alleviating median nerve compression and associated symptoms. The advent of open carpal tunnel release established surgery as the definitive treatment for moderate-to-severe CTS, with subsequent refinements—such as mini-open and endoscopic techniques—focused on minimizing tissue trauma and expediting recovery. Comparative studies demonstrate similar long-term efficacy between surgical modalities, though endoscopic approaches often provide faster short-term recovery. Advances in diagnostic imaging, including high-resolution ultrasound, have improved early detection and dynamic assessment of median nerve compression. Emerging therapies, such as regenerative biologics, neuromobilization, and minimally invasive surgical innovations, offer promising adjuncts to current care. Despite substantial progress, further research is needed to clarify optimal patient selection, refine minimally invasive techniques, and explore regenerative interventions. This review underscores the importance of individualized, evidence-based, and patient-centered approaches to CTS management, integrating both established and emerging strategies to optimize functional outcomes and quality of life.

## 1. Introduction

Carpal tunnel syndrome (CTS), a prevalent peripheral nerve compression disorder, has garnered significant attention in both medical literature and the public sphere—particularly since its recognition as a work-related injury in the 1980s [1]. Epidemiological estimates suggest that roughly one in ten individuals will experience CTS at some point in their lives, underscoring its widespread impact [2].

CTS arises from entrapment and compression of the median nerve within the carpal tunnel at the wrist, making it the most frequently encountered mononeuropathy in clinical practice [3]. The carpal tunnel itself is a narrow anatomical passageway bounded by the carpal bones on three sides and the transverse carpal ligament on the palmar aspect [4]. The median nerve traverses this confined space alongside the flexor tendons that facilitate finger and thumb flexion [5].

Compression of the median nerve within the rigid tunnel leads to the classic symptoms of CTS: pain, numbness, tingling, and weakness in the hand and fingers, particularly affecting the thumb, index, middle, and radial half of the ring finger. Given the debilitating nature of CTS and its potential to significantly impair hand function and quality of life, a comprehensive understanding of its etiology, diagnosis, and management is paramount for healthcare professionals. The evolution of CTS treatment strategies over time reflects ongoing advancements in medical knowledge, diagnostic modalities, and surgical techniques—all aimed at improving patient outcomes and minimizing the burden of this common condition.

Over the years, the understanding and management of CTS have undergone significant evolution, marked by improvements in both conservative care and surgical intervention. Initially, treatment strategies centered around conservative measures designed to alleviate pressure on the median nerve and reduce inflammation [6]. Patients typically experience a gradual onset of symptoms that often worsen at night (nocturnal exacerbation) and can radiate up the forearm; repetitive hand movements or sustained wrist flexion/extension frequently aggravate the condition [7]. Early management therefore included rest and immobilization of the wrist, often with the use of wrist splints to maintain a neutral position, as well as adjunctive therapies like heat or cold application for symptom relief Nonsteroidal anti-inflammatory drugs (NSAIDs) were commonly used to manage pain and inflammation, and corticosteroid injections into the carpal tunnel emerged as a non-surgical option that could provide temporary relief by reducing swelling around the median nerve. While these conservative approaches often achieved short-term symptom alleviation, their long-term efficacy was variable, and many patients eventually required surgical intervention to obtain lasting relief [7]. Notably, CTS is recognized as the most commonly diagnosed entrapment neuropathy of the upper extremity [8] and the most prevalent peripheral compressive neuropathy overall [9], which underscores the importance of understanding its pathophysiology and optimizing treatment strategies.

Surgical management of CTS has likewise evolved substantially. Historically, the primary surgical treatment was an open carpal tunnel release, performed through a relatively large incision to fully divide the transverse carpal ligament and decompress the median nerve [10]. Open release became the gold standard due to its high success rates in resolving symptoms and restoring hand function. As surgical techniques and instruments advanced, however, there was a shift toward minimizing tissue trauma in order to reduce postoperative pain and speed recovery. This led to the development of less invasive approaches—most notably, endoscopic carpal tunnel release, which uses much smaller incisions and fiber-optic visualization to cut the transverse ligament. The endoscopic approach has offered advantages such as reduced postoperative discomfort, faster return to normal activities, and a smaller scar, albeit with a steeper learning curve for surgeons and some concerns about complications (e.g., risk of nerve injury or incomplete release of the ligament). Overall, long-term outcome studies have shown that the majority of patients report good to excellent results after carpal tunnel release surgery, with significant improvement in symptoms and hand function [11].

This narrative review aims to provide a comprehensive overview of the evolution of carpal tunnel syndrome management, tracing the historical progression of its diagnosis and treatment and highlighting key advancements in the field. We begin with the early recognition of CTS as a distinct clinical entity and the initial approaches to treatment, then discuss the refinement of diagnostic criteria alongside developments in electrophysiological testing and imaging. We explore the evolution of surgical techniques—from the traditional open carpal tunnel release to minimally invasive endoscopic procedures—underscoring the advantages and drawbacks of each. Advancements in non-surgical management are also reviewed, including modern physical therapy modalities and ergonomic interventions. Finally, we compare the outcomes of surgical versus conservative treatments, examine common complications and their prevention, and consider future directions and emerging therapies in CTS care.

## 2. Materials and Methods

A thorough literature review was conducted to gather relevant information for this narrative review of carpal tunnel syndrome. The search strategy included a combination of keywords related to carpal tunnel syndrome, its etiology, diagnosis, treatment, and surgical techniques. Relevant articles were identified through electronic databases such as PubMed and Scopus, covering sources from the earliest documented descriptions of carpal tunnel syndrome to the most recent advances in its management. The terms used were ‘carpal tunnel syndrome,’ ‘CTS,’ ‘median nerve compression,’ ‘splinting,’ ‘corticosteroid injection,’ ‘physical therapy,’ ‘endoscopic release,’ ‘ultrasound,’ and ‘regenerative therapy,’ covering publications up to December 2024. Inclusion criteria were CTS-focused clinical or anatomical relevance; exclusion criteria were non-CTS neuropathies, basic science without clinical translation, and meta-analyses unrelated to CTS. Inclusion criteria encompassed publications focusing on the pathophysiology, clinical presentation, diagnostic methods, conservative treatments, surgical interventions, and outcomes of carpal tunnel syndrome, with no restrictions on study design or year of publication. Data from each selected article were extracted regarding study design, sample characteristics, diagnostic criteria, treatment methods, outcome measures, and any reported complication rates. The extracted information was then synthesized to construct a comprehensive overview of the evolution of carpal tunnel syndrome management, emphasizing key milestones, emerging challenges, and ongoing controversies in the field. Articles were ultimately chosen based on their relevance to the topic and their contribution to the current understanding of carpal tunnel syndrome management.

## 3. Results and Discussion

### 3.1. Historical Perspective on Carpal Tunnel Treatment

#### 3.1.1. Early Recognition and Initial Approaches

The earliest documented descriptions of carpal tunnel syndrome date back to the mid-19th century, although the condition was not fully characterized as a distinct clinical entity until well into the 20th century. Before CTS was formally recognized, patients presenting with hand pain, numbness, and weakness were often misdiagnosed or had their symptoms attributed to other conditions such as arthritis or cervical radiculopathy. In the early 20th century, as medical understanding advanced, clinicians began to identify a characteristic pattern of symptoms associated with compression of the median nerve at the wrist. Initial treatment approaches were largely conservative and symptomatic, focused on relieving discomfort and reducing inflammation. These early measures included prescribed rest for the affected hand, immobilization of the wrist using splints, and the application of heat or cold therapy to alleviate symptoms [12].

#### 3.1.2. Formal Recognition and Early Surgical Management

Carpal tunnel syndrome was formally defined and popularized as a specific diagnosis in the mid-20th century, largely through the work of Dr. George S. Phalen. Phalen published a series of influential papers describing the characteristic symptoms and physical exam findings (such as the wrist flexion test that now bears his name) indicative of median nerve compression at the wrist. His contributions were pivotal in establishing CTS as a well-defined clinical syndrome and laid the groundwork for targeted treatment strategies. During this period, the pathophysiology of CTS became better understood—particularly the role of the transverse carpal ligament in constricting the carpal tunnel and compressing the median nerve. Early surgical interventions for CTS emerged around this time, primarily involving an open release of the transverse carpal ligament to decompress the median nerve. These initial carpal tunnel release surgeries were performed through relatively large incisions in the palm, allowing direct visualization of the ligament and nerve. While often effective in alleviating symptoms, the procedures were invasive, and patients commonly experienced significant postoperative pain and lengthy recovery periods [13].

#### 3.1.3. Development of Surgical Techniques

The open carpal tunnel release technique described above quickly became the standard surgical approach for moderate to severe CTS, owing to its reliable symptom relief and improvement in hand function. As surgical knowledge and technology advanced in the latter half of the 20th century, surgeons sought to refine this procedure to reduce patient morbidity. The focus shifted toward minimizing soft tissue disruption, decreasing postoperative pain, and accelerating the return to normal activities. The advent of minimally invasive surgical techniques for CTS marked a pivotal turning point in this evolution, offering the promise of reduced surgical trauma and quicker rehabilitation for patients [13].

#### 3.1.4. Introduction of Conservative Treatments

Conservative (non-surgical) treatments have long played a significant role in the management of carpal tunnel syndrome, especially for patients with mild to moderate symptoms or those seeking to avoid surgery. Historically, a variety of non-surgical interventions have been employed:

- Wrist splinting: Rigid wrist splints are often worn at night to keep the wrist in a neutral position, thereby reducing pressure on the median nerve during sleep and alleviating nocturnal symptoms.
- Nonsteroidal anti-inflammatory drugs (NSAIDs): Medications such as ibuprofen are used to manage pain and reduce inflammation in and around the carpal tunnel.
- Corticosteroid injections: Injecting a corticosteroid (a potent anti-inflammatory agent) directly into the carpal tunnel can provide temporary relief by diminishing swelling around the median nerve [14].
- Physical therapy: Therapeutic exercises and modalities have been incorporated to improve wrist and hand function. These include strength and flexibility exercises, nerve gliding techniques to enhance median nerve mobility, and ergonomic modifications to reduce strain on the wrist [15].

Each of these conservative strategies can be beneficial in the short to medium term. Wrist splinting, in particular, became a cornerstone of early CTS management due to its simplicity and efficacy in many cases. Corticosteroid injections often yield significant but transient symptom relief and can also serve as a diagnostic tool—a positive response to an injection tends to support the diagnosis of CTS [15]. It is important to note that while conservative treatments may effectively manage symptoms for a time, they do not permanently resolve the underlying issue of median nerve compression. As a result, patients with persistent or severe symptoms frequently progress to surgical intervention for definitive treatment. Current recommendations from the AAOS Clinical Practice Guideline (2024) were incorporated to strengthen evidence-based discussion [16].

### 3.2. Evolution of Surgical Techniques

#### 3.2.1. Open Carpal Tunnel Release

Open carpal tunnel release is the traditional surgical approach for CTS and has been performed for many decades with generally excellent outcomes. In an open release, the surgeon makes an incision (usually 3–5 cm long) in the palm of the hand along the axis of the ring finger, allowing direct access to the transverse carpal ligament. The ligament is then carefully divided to relieve pressure on the underlying median nerve [17]. This method allows for thorough visualization of the anatomy, enabling the surgeon to identify anatomical variations or contributing factors (such as tenosynovitis of the flexor tendons) that might also be addressed during the procedure. Open release surgery boasts high success rates in terms of symptom relief and functional recovery. However, its drawbacks include the relatively large incision and the associated scar, a higher degree of postoperative pain and tenderness around the scar, and a longer healing time compared to newer, less invasive techniques.

#### 3.2.2. Mini-Open Carpal Tunnel Release

The mini-open carpal tunnel release is a refinement of the open technique that uses a smaller incision while still allowing the surgeon to directly visualize and cut the transverse carpal ligament. In this approach, a limited incision (often around 1.5–2 cm) is made at the base of the palm or at the wrist crease. Through this smaller portal, the surgeon can insert specialized instruments to divide the ligament under direct vision. The goal of the mini-open approach is to minimize soft tissue dissection and trauma, thereby potentially reducing postoperative pain and expediting the patient’s recovery, while maintaining the precision and completeness of a standard open release [18]. The mini-open technique strikes a balance between the familiarity and direct control of the open procedure and the reduced invasiveness of endoscopic methods. Patients who undergo mini-open release may experience faster return to daily activities and report high satisfaction rates, although outcomes are generally comparable to the classic open surgery when both are performed effectively.

#### 3.2.3. Endoscopic Carpal Tunnel Release

Endoscopic carpal tunnel release is a minimally invasive alternative to the open procedure, introduced in the late 20th century as surgical technology advanced. In endoscopic release, one or two very small incisions (typically about 1 cm each) are made in the wrist or palm. Through these incisions, a tiny endoscope (camera) and specialized cutting tools are inserted. The endoscope provides internal visualization of the carpal tunnel on a video monitor, guiding the surgeon as they cut the transverse carpal ligament from beneath [19]. By avoiding a large open incision, the endoscopic approach generally causes less tissue disruption, which can translate to reduced postoperative pain, less scar sensitivity, and a quicker recovery for the patient [20].

There are two main techniques for endoscopic release: the single-portal and the two-portal methods. In the single-portal technique, all instruments (camera and cutter) are introduced through one incision, usually at the wrist. In the two-portal technique, one incision is used for the endoscope and a separate incision for the cutting instrument. Both methods aim to achieve the same result—complete division of the transverse carpal ligament—yet each has its nuances. The single-portal approach creates only one scar and is slightly less invasive, whereas the two-portal approach can sometimes provide better instrument maneuverability and visualization by separating the entry points. Importantly, the primary surgical goal, whether using open or endoscopic methods, remains the full release of the transverse carpal ligament to ensure adequate decompression of the median nerve [21]. Throughout endoscopic procedures, maintaining clear visualization of the median nerve and surrounding structures is critical, as the confined working space and indirect viewing can increase the risk of accidental nerve or tendon injury if not performed with care [22].

Overview of surgical CTS techniques is given in Table 1.

### 3.3. Comparative Studies and Outcomes

A substantial body of research, including randomized trials and meta-analyses, has compared the outcomes of open versus endoscopic (and more recently mini-open) carpal tunnel release to determine the optimal surgical approach. Many studies indicate that endoscopic release offers certain short-term advantages over the open technique—specifically, patients often experience less postoperative pain, a faster recovery of grip strength, and an earlier return to work and daily activities following endoscopic surgery. On the other hand, numerous investigations have found no significant differences in long-term results between endoscopic and open releases in terms of symptom relief, nerve functional recovery, and patient satisfaction once healing is complete. This suggests that, in the long run, both techniques are highly effective and the choice may depend on the surgeon’s expertise and the patient’s specific situation [23]. Surgeons typically consider factors such as the severity of nerve compression, the patient’s anatomy (e.g., wrist size, any anatomic anomalies), and any coexisting conditions (like inflammatory arthritis or diabetes) when deciding which surgical method to use for a given patient.

Beyond technique comparisons, research has also explored the biomechanics of carpal tunnel release. For instance, studies have examined changes in carpal tunnel pressure and compliance when the flexor retinaculum (transverse ligament) is incrementally released, to better understand how much division is necessary to achieve adequate decompression [24]. Overall, the evidence confirms that complete release of the ligament is crucial; incomplete release can lead to persistent or recurrent symptoms, whereas an excessive release or unnecessary additional dissection could contribute to instability or other complications. Continued innovations in surgical instruments and technique refinement, along with high-quality comparative studies, will further clarify how to optimize outcomes for CTS patients while minimizing risks.

### 3.4. Advancements in Non-Surgical Management

#### 3.4.1. Splinting and Bracing

Splinting and bracing are foundational components of non-surgical management for carpal tunnel syndrome. The primary objective of wrist splinting is to immobilize the wrist in a neutral (or slightly extended) position, thereby maximizing the volume of the carpal tunnel and minimizing pressure on the median nerve. Patients are often advised to wear wrist splints at night because CTS symptoms frequently worsen during sleep due to inadvertent wrist flexion. By keeping the wrist straight, night splints can substantially reduce nocturnal numbness and pain. Braces serve a similar purpose and may be used during the day for certain activities; they tend to be more adjustable and can offer support while still permitting some degree of hand movement. The effectiveness of splinting and bracing can vary between individuals and typically correlates with the severity of CTS and duration of use. For milder cases or early-stage CTS, consistent use of a well-fitting splint often provides significant symptom relief. However, patient compliance is crucial—splints must be worn regularly (especially at night) and fit properly to achieve the desired benefit. Educating patients on the correct way to apply and wear their splint, as well as when to use it, is an important part of treatment to ensure compliance and optimize outcomes [25].

#### 3.4.2. Corticosteroid Injections

Corticosteroid injections into the carpal tunnel are a commonly employed conservative treatment for CTS, offering temporary symptom relief by combating local inflammation (a non-steroidal alternative increasingly studied is 5% dextrose (D5W) perineural injection, offering neuroregenerative and anti-inflammatory benefit without steroid-related risks). Corticosteroids are powerful anti-inflammatory medications that, when injected around the median nerve, can reduce swelling of the tendon sheaths and other tissues within the carpal tunnel. This reduction in inflammation can lead to an appreciable decrease in pain, as well as improvements in numbness and tingling in the weeks following the injection. The injection procedure is typically performed in an outpatient setting under local anesthesia or even without it, as it only takes a few minutes and is generally well tolerated by patients. Ultrasound guidance improves needle accuracy, reduces iatrogenic contact with the median nerve, and enhances safety. Ultrasound-guided transverse carpal ligament (TCL) release is an emerging minimally invasive alternative that allows precise decompression under real-time visualization. Symptom relief from a steroid injection can vary widely in duration; some patients may experience improvement for only a few weeks, while others might have relief for several months. Steroid injections can also play a diagnostic role: if symptoms are significantly improved after an injection, it provides supporting evidence that the diagnosis of CTS is correct (since relieving pressure/inflammation in the carpal tunnel directly led to symptom reduction) [15]. It is important to note that while injections can be repeated, there are limits due to potential side effects (such as tissue atrophy or tendon weakening), and repeated injections may have diminishing returns. Therefore, corticosteroid injections are often used as an interim measure or for patients who either are not immediate surgical candidates or prefer to delay surgery.

### 3.5. Physical Therapy and Ergonomic Modifications

Physical therapy (PT) interventions and ergonomic modifications constitute a critical aspect of comprehensive CTS management, particularly for patients seeking to avoid or postpone surgery. Physical therapy approaches for CTS are multifaceted [26]. A therapist may employ manual therapy techniques—such as carpal bone mobilization and soft tissue mobilization of the forearm musculature—to reduce restrictions and improve the mechanical environment of the carpal tunnel, thereby improving overall wrist and hand function [27]. Nerve gliding (or neural mobilization) exercises are commonly taught to patients; these exercises gently move the median nerve through its normal range within the carpal tunnel and distal extremity, which can help maintain nerve mobility and reduce adhesions. Additionally, targeted stretching and strengthening exercises for the hand, wrist, and forearm muscles are implemented to address any muscular imbalances and improve overall support to the wrist joint.

Various therapeutic modalities have also been explored to alleviate CTS symptoms. For example, the use of therapeutic ultrasound and low-level laser therapy on the carpal tunnel region has shown some benefit in reducing pain and promoting nerve healing in the short term [27]. Additional modalities widely used in clinical practice include enhanced neuromobilization, hydrodissection/hydrodecompression, carpal bone and soft tissue mobilization, Kinesio Taping (KT), all of which demonstrate adjunctive symptom relief in mild–moderate CTS. Wrist orthoses (splints) can be custom-fabricated by therapists to ensure an optimal fit, and patients are educated in activity modifications to avoid positions or motions that exacerbate their symptoms. Ergonomic adjustments are especially relevant for work-related CTS: this can involve evaluating and modifying a patient’s workspace or tool use (keyboard height, mouse design, vibration reduction in power tools, etc.) to minimize sustained wrist flexion or extension and excessive repetitive hand movements.

Evidence supporting these conservative therapies indicates that they can offer moderate improvement in symptoms for mild-to-moderate CTS, at least in the short to medium term [27]. For instance, a combination of splinting, exercise, and manual therapy might significantly reduce a patient’s nighttime numbness and improve grip strength over a period of weeks to months. However, the degree of benefit is variable, and such measures are often less effective if nerve compression is severe or long-standing (where surgical decompression might be more definitively corrective). Nonetheless, physical therapy and ergonomic interventions are widely used due to their non-invasive nature, relative affordability, and the empowerment they give patients in self-managing their condition.

Overview of conservative CTS techniques is given in Table 2.

### 3.6. Emerging Therapies and Future Directions

#### 3.6.1. Ultrasound Therapy and Other Novel Treatments

Emerging treatment modalities for carpal tunnel syndrome are being investigated as alternatives or adjuncts to standard care. One such modality is therapeutic ultrasound, which uses high-frequency sound waves applied to the carpal tunnel region. The mechanical and thermal effects of ultrasound therapy may promote tissue healing, reduce inflammation, and improve median nerve function, although clinical evidence is still accumulating. Interestingly, ultrasound is also proving valuable as a diagnostic and monitoring tool: high-resolution ultrasound imaging can visualize the median nerve within the carpal tunnel and detect changes in its cross-sectional area or movement with various wrist positions [29]. This can help clinicians assess the severity of nerve compression and the effect of dynamic movements on the nerve, information that can guide treatment decisions.

Beyond ultrasound, researchers are exploring biologic therapies for CTS. For instance, platelet-rich plasma (PRP) injections, which involve injecting a concentrated sample of the patient’s own platelets and growth factors into the carpal tunnel, have been studied for their potential to reduce inflammation and encourage tissue repair. Similarly, preliminary investigations into stem cell injections aim to determine if regenerative cells can reverse nerve damage or promote healing of the median nerve in chronic cases of CTS.

Another innovative approach in conservative management is enhanced neuromobilization techniques. Neuromobilization refers to specialized exercises that improve the mobility and gliding of peripheral nerves. When added to a standard physical therapy regimen, these advanced nerve gliding techniques have shown promise in further improving symptoms and functional outcomes for CTS patients. The integration of such exercises can be particularly useful for patients who have residual symptoms despite other treatments, as improving nerve dynamics might address subtler components of nerve entrapment.

#### 3.6.2. Technological Innovations in Diagnosis and Treatment

Technological advancements are continually informing the diagnosis and treatment of carpal tunnel syndrome. Researchers are working on developing more sensitive and specific diagnostic tools that can detect CTS changes earlier or distinguish it from other conditions with similar symptoms. For example, nerve conduction studies are a current diagnostic standard, but newer techniques like high-frequency ultrasound imaging (as mentioned above) provide a non-invasive and real-time way to evaluate nerve compression, still nerve conduction studies (NCS/ENG) and electromyography (EMG) remain the gold standard for confirming severity and differentiating CTS from cervical radiculopathy or polyneuropathy.

Ultrasound, in fact, has been noted to be more comfortable for patients, less time-consuming, and less expensive than MRI for imaging the carpal tunnel—while still achieving comparable accuracy in the hands of an experienced examiner [30]. As this technology becomes more widespread, it may become a first-line diagnostic adjunct alongside clinical examination.

On the treatment front, innovation is leading to improved medical devices and materials. For instance, there is ongoing development of more ergonomic splints and braces using lightweight, breathable materials that increase patient comfort and compliance. In surgery, new endoscopic instruments and visualization tools are being refined to make minimally invasive techniques safer and more effective. Additionally, researchers are investigating robotic-assisted surgical systems for hand and wrist surgery, which could potentially improve precision in delicate operations like carpal tunnel release.

Future directions in CTS management also include personalized medicine approaches. Scientists are exploring the role of genetics and systemic factors in predisposing individuals to CTS, which might one day allow for risk stratification and preventive strategies in high-risk populations. Efforts are underway to identify molecular or imaging biomarkers that could signal early median nerve distress before irreversible damage occurs, thereby enabling earlier intervention (perhaps even before symptoms become severe). Despite these high-tech developments, it is worth emphasizing that fundamental conservative measures remain highly relevant—physical therapy interventions and ergonomic changes are widely utilized because they are non-invasive, cost-effective, and accessible approaches that can benefit a large number of patients [31]. The challenge and opportunity moving forward lie in integrating these traditional therapies with new technologies to create more effective, patient-tailored treatment plans.

### 3.7. Comparative Analysis of Treatment Modalities

#### 3.7.1. Efficacy of Surgical vs. Non-Surgical Interventions

**Figure 1:**
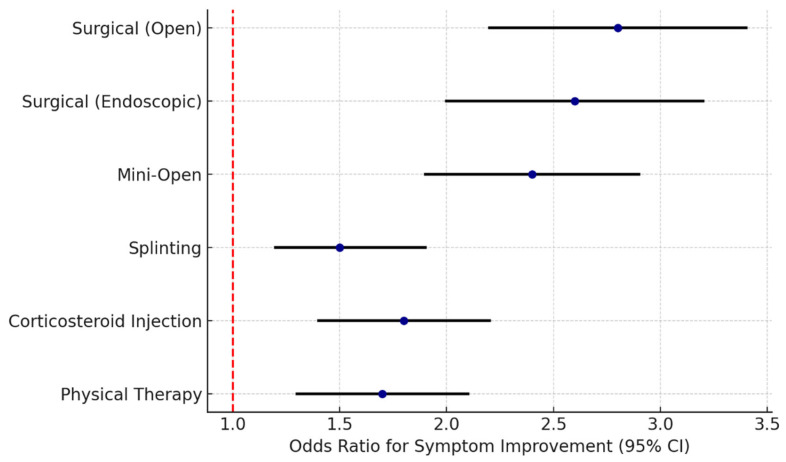
Comparative Outcomes of CTS Treatment Modalities. (Data synthesized from peer-reviewed CTS studies. Refs. [13,14,15,16,17,18,19,20,21,22,23,24,25,26,27,28,29,30,31,32,33,34].)

When evaluating treatment options for carpal tunnel syndrome, a key consideration is the relative efficacy of surgical versus non-surgical interventions (Figure 1). Surgical treatment (typically carpal tunnel release surgery) directly addresses the cause of CTS by cutting the transverse carpal ligament to relieve pressure on the median nerve. In contrast, non-surgical treatments (such as splinting, medications, injections, and therapy) aim to manage symptoms and improve hand function without altering the anatomy of the carpal tunnel. The decision to pursue conservative management or proceed to surgery depends on multiple factors, including the severity and duration of symptoms, the degree of median nerve impairment on clinical and electrodiagnostic testing, patient preferences, and any contraindications. For patients with mild symptoms, a trial of conservative treatment—encompassing activity modifications, intermittent wrist exercises, splint use, and other pain-relieving modalities—can be quite useful and may even be enhanced by complementary practices like yoga for hand and wrist flexibility [32]. However, if a patient’s symptoms are worsening or fail to improve after an adequate course of non-surgical therapy, surgical intervention is generally recommended to prevent further nerve damage and permanently resolve the compression.

#### 3.7.2. Long-Term Outcomes and Recurrence Rates

Long-term outcomes and the risk of symptom recurrence are important metrics when comparing treatment modalities for CTS. Many studies suggest that surgical intervention provides more definitive and longer-lasting relief than conservative measures, particularly for moderate to severe CTS. Carpal tunnel release surgery often leads to substantial and sustained symptom improvement, but it is not universally curative. Recurrence of CTS symptoms or the need for a repeat surgery (revision release) can occur in a subset of patients, with reported recurrence rates varying widely depending on the length of follow-up and the definitions used [33]. Factors such as incomplete initial release of the ligament, scar tissue formation, or underlying conditions (like diabetes or thyroid disorders that predispose to neuropathy) can contribute to recurrent symptoms post-surgery. Conversely, nonsurgical treatments tend to have more modest long-term outcomes. While splinting, injections, and therapy can significantly alleviate symptoms in the short term, their benefits often diminish over time if the precipitating activities are resumed or if the underlying compression remains unaddressed. In chronic or severe cases left untreated surgically, there is a risk of permanent nerve damage, which underscores the importance of timely intervention. Ultimately, the best long-term results are seen when the chosen therapy is appropriately matched to the severity of the condition: mild cases can often be managed successfully without surgery, but long-standing or severe CTS frequently requires surgical decompression for a lasting solution.

#### 3.7.3. Patient Satisfaction and Quality of Life

Patient satisfaction and quality of life are critical considerations in judging the success of any CTS treatment. Because CTS can significantly impair daily activities (from buttoning a shirt to holding a pen or typing on a keyboard), effective treatment should not only alleviate clinical symptoms but also restore the patient’s ability to function comfortably in day-to-day life. In general, patients who undergo treatment—whether conservative or surgical—experience improvements in pain, hand strength, dexterity, and sleep quality once their CTS is addressed. Multiple studies have found that surgical treatment yields high patient satisfaction rates, especially in cases of severe CTS where surgery often produces dramatic relief of numbness and nocturnal pain. These patients frequently report substantial gains in quality of life after recovering from surgery, as they can return to work or hobbies that were limited by their hand symptoms. It has been observed that satisfaction levels tend to be highest when symptoms are fully or nearly fully resolved, which surgery is more likely to achieve in advanced cases.

However, non-surgical treatments also play an important role and can meaningfully improve quality of life for many individuals. For those with mild-to-moderate CTS or those who cannot undergo surgery for health or personal reasons, conservative measures that reduce pain and tingling can lead to better sleep, improved hand function, and an overall sense of well-being. Although the symptom relief from these measures may not be complete or permanent, patients often appreciate the avoidance of surgery and its associated costs and risks. It is also worth noting that because CTS is the most common and widely researched compressive neuropathy, there are well-established protocols and a breadth of resources available to support patients through whichever treatment path they choose [34]. In modern practice, shared decision-making is emphasized: healthcare providers discuss the expected outcomes, limitations, and potential risks of each approach with patients, allowing them to weigh these factors in light of their personal values and lifestyle. The ultimate goal is to ensure that treatment not only improves clinical measures but also aligns with the patient’s expectations and enhances their overall quality of life.

### 3.8. Complications and Management Strategies

#### 3.8.1. Surgical Complications and Their Management

While carpal tunnel release surgery is generally safe and highly effective, as with any surgical procedure it carries certain risks of complications [35]. Common complications of carpal tunnel surgery include:

- Infection: Though infrequent, post-surgical wound infections can occur. This risk is minimized by adhering to strict sterile techniques in the operating room and proper wound care after surgery. Minor infections can often be managed with oral antibiotics, whereas deeper infections may require intravenous antibiotics or, rarely, surgical drainage.
- Nerve injury: Intraoperative injury to the median nerve or its branches (such as recurrent motor branch of the median nerve, which innervates the thenar muscles) is a serious but rare complication. An injured nerve can lead to persistent numbness, loss of function, or neuropathic pain. Preventive measures include careful surgical technique and clear identification of anatomical landmarks; if an injury is recognized, nerve repair may be attempted.
- Hematoma formation: Bleeding at the surgical site can lead to a hematoma (a localized collection of blood) within the carpal tunnel or under the incision. Hematomas can cause swelling, pain, and potentially pressure on the nerve. Managing this may involve elevating the hand, applying ice, and sometimes returning to the operating room to evacuate the hematoma if it is large or compromising circulation.
- Scar tenderness: After an open carpal tunnel release, it is not uncommon for patients to experience tenderness or hypersensitivity at the scar on the palm. This usually improves over time. Management includes desensitization techniques (gentle massage, texture exposure), silicone gel pads, or steroid injections into the scar if the sensitivity is pronounced. Keeping the scar supple with massage and avoiding pressure on the heel of the hand during early recovery can help reduce scar discomfort.

Surgeons are vigilant about these potential complications and take steps to mitigate them. For example, prophylactic antibiotics might be given to reduce infection risk, and careful intraoperative hemostasis (controlling bleeding) is maintained to prevent hematoma. Overall, the incidence of serious complications from carpal tunnel surgery is low, and most issues that do arise are manageable with appropriate medical or surgical intervention.

#### 3.8.2. Complications Related to Non-Surgical Treatments

Non-surgical treatments for CTS are generally low-risk, but they are not entirely free of potential complications or side effects. Some of the considerations include:

- Corticosteroid injections: While often helpful for temporary relief, steroid injections can cause localized complications such as pain at the injection site, swelling, or even transient discoloration of the skin. In some cases, patients might experience a “cortisone flare” (an acute increase in pain due to crystallization of the steroid) for a day or two after the shot. Repeated injections carry a small risk of damage to structures within the carpal tunnel, and too-frequent steroid use can weaken nearby tendons or cause thinning of the skin and subcutaneous tissues.
- Extended splint use: Using a wrist splint continuously for long periods (beyond just night use or strategic daytime use) can lead to joint stiffness and reduced range of motion in the wrist. Muscular effects are also a concern—prolonged immobilization may result in weakening of the hand and forearm muscles and even atrophy from disuse [36]. It is important that patients balance rest with regular gentle movement exercises to keep the joints flexible and muscles conditioned.
- Over-aggressive physical therapy: While therapeutic exercises are usually beneficial, if they are performed incorrectly or too vigorously, they can exacerbate CTS symptoms. For example, overly strenuous wrist stretches or strengthening exercises performed in the midst of active nerve compression could increase inflammation or further irritate the median nerve. It is crucial that any exercise regimen for CTS be guided by a knowledgeable professional and tailored to the patient’s tolerance.

Monitoring and patient education are key to minimizing these conservative-treatment-related issues. Patients receiving a corticosteroid injection are typically warned about and monitored for any adverse reactions. Those using splints are advised on proper timing (e.g., mostly during sleep) and the importance of regular movement breaks. Physical therapists instruct patients on pain signals and correct form to ensure that exercises help rather than harm. By staying attuned to the patient’s response and adjusting the treatment plan as needed, healthcare providers can manage minor complications of non-surgical therapy effectively and ensure a safe course of conservative management.

### 3.9. Strategies for Preventing Complications

Preventing complications in the management of carpal tunnel syndrome involves proactive strategies in both surgical and non-surgical contexts:

- Meticulous surgical technique: For those undergoing carpal tunnel release, the surgeon’s skill and attention to detail are paramount. Identifying anatomical landmarks, using gentle tissue handling, and ensuring complete release of the transverse ligament while avoiding excessive dissection help prevent many surgical complications. Adhering strictly to sterile protocols also reduces infection risk.
- Patient selection and preoperative planning: Good outcomes begin with appropriate patient selection. Surgeons evaluate whether symptoms correlate with objective findings and confirm the diagnosis (often with nerve conduction studies) to ensure surgery is warranted. Any medical comorbidities (like coagulopathies or uncontrolled diabetes) are addressed preoperatively to decrease the risk of complications such as bleeding or poor wound healing.
- Postoperative care and rehabilitation: Effective pain management and guided rehabilitation after surgery can significantly influence recovery. Elevating the hand, using ice, and taking prescribed pain medications in the immediate postoperative period help control swelling and discomfort, potentially preventing complications like excessive scar adhesion or stiffness. Early mobilization of the fingers and thumb is encouraged while protecting the healing incision, to maintain tendon gliding and circulation. Structured hand therapy, when needed, can assist in scar management and restoring strength safely.
- Education for conservative management: When treating CTS non-surgically, educating patients is essential for complication prevention. Patients should understand how to properly wear and not overuse wrist splints, how to perform exercises with correct technique, and how to listen to their symptoms to avoid overexertion. Follow-up appointments are useful for reinforcing these points and for monitoring progress, ensuring that interventions are helping and not inadvertently causing harm.
- Use of established best practices: In navigating newer treatment options, it is often safest to rely on techniques and therapies with proven track records. For example, open carpal tunnel release is a well-established procedure with predictable outcomes and is often considered the gold standard against which newer techniques are measured [14]. By using validated methods (or adopting new ones only after sufficient evidence of safety and efficacy), clinicians can minimize unforeseen complications.

Through these strategies—careful surgery, prudent patient management, and ongoing education—most potential complications of CTS treatment can be either prevented or promptly identified and addressed, thereby maintaining a high level of safety in the care of this common condition.

## 4. Conclusions

### 4.1. Summary of the Evolution of Carpal Tunnel Treatment

Over the past century, the management of carpal tunnel syndrome has seen significant evolution, reflecting broader advancements in medical science and technology. Initially, treatment was predominantly conservative, with an emphasis on rest, splinting, and simple remedies aimed at symptom relief. As the condition became better defined and understood (thanks in part to pioneers like Phalen), surgical intervention in the form of open carpal tunnel release became a mainstay, offering definitive relief for many sufferers. Subsequent refinements in surgical technique—culminating in the development of endoscopic and other minimally invasive approaches—have sought to reduce the recovery burden while maintaining the high success rates of traditional surgery. Non-surgical care has likewise progressed, integrating a greater role for patient education, ergonomic adjustments, and interdisciplinary rehabilitation. Overall, the trajectory of CTS treatment highlights a trend toward individualized care and shared decision-making, acknowledging that one size does not fit all. Clinicians now have an array of tools at their disposal, and the challenge is to apply the right intervention, or combination of interventions, for the right patient at the right time [13].

### 4.2. Future Directions in Carpal Tunnel Management

Looking ahead, the field of carpal tunnel syndrome management is poised to benefit from ongoing research and innovation. Future directions include the development of more effective non-surgical therapies that could delay or obviate the need for surgery in certain patients, as well as further refinement of surgical techniques to make them less invasive and more efficient. For instance, the trend toward minimally invasive surgery continues—“keyhole” or single-incision carpal tunnel release techniques are becoming more widely accepted as surgeons gain experience with them [37]. There is also a growing interest in the use of biomarkers and advanced imaging to identify patients at risk of developing CTS or to diagnose it earlier in its course. By recognizing the condition sooner, interventions can be implemented before significant nerve damage occurs. On the surgical front, emerging technologies such as high-definition endoscopic cameras, improved cutting tools, and even robot-assisted microsurgery hold the promise of enhancing precision and patient outcomes. Additionally, research into the genetic and molecular underpinnings of CTS may reveal insights that lead to novel preventative strategies or treatments—perhaps specific medications that can be used to treat underlying tendon inflammation or nerve ischemia. The ultimate goal for future management is to provide personalized therapy that maximizes symptom resolution and functional recovery for each individual patient [38].

### 4.3. Current Best Practices

Current best practices for CTS emphasize an evidence-based, patient-centered approach. In most cases, a trial of conservative management is recommended for patients with mild to moderate symptoms. This typically includes wrist splinting (especially at night), activity modifications to reduce wrist strain, and possibly a corticosteroid injection or a course of physical therapy [39]. Such measures can effectively alleviate symptoms in a significant number of cases. Early diagnosis and intervention are crucial: recognizing CTS early and implementing these measures can prevent permanent median nerve damage and improve the likelihood of full recovery [40]. If a patient does not respond to conservative treatment, or if they present with severe symptoms (such as constant numbness, thenar muscle atrophy, or markedly abnormal nerve conduction studies), surgical release of the carpal tunnel is indicated. Both open and endoscopic carpal tunnel release procedures are widely practiced, and each has specific indications and considerations. Open release remains a highly reliable option, while endoscopic release offers the benefit of faster postoperative recovery for some patients. It is important for clinicians to weigh the risks and benefits of each approach; for example, the risk factors for complications and the rates of any necessary revision surgery differ slightly between open and endoscopic techniques [41,42]. In essence, the current standard of care is to tailor treatment intensity to disease severity and patient needs—applying the least invasive yet effective option and escalating to surgical intervention when appropriate—to achieve the best functional outcome with the least risk.

### 4.4. Future Research Needs and Opportunities

Several key areas have been identified where further research could substantially impact the management of carpal tunnel syndrome:

1. Comparative effectiveness studies: Large-scale, long-term studies comparing surgical and non-surgical treatments (including newer interventions) are needed to determine which approaches yield the best outcomes for different patient subsets. These studies would inform guidelines on when to opt for surgery versus continued conservative care.
2. Biomechanical insights: More research into the biomechanical and ergonomic factors that contribute to CTS could lead to improved preventive measures. Understanding how specific repetitive movements, wrist postures, or tool designs increase carpal tunnel pressure could drive the creation of better workplace ergonomics and protective equipment to reduce incidence.
3. Predictors of treatment success: Investigations aimed at identifying which patient-specific factors (e.g., genetic markers, coexisting conditions, or imaging findings) predict a better response to certain treatments would enable personalized treatment plans. For instance, if certain imaging features predict poor response to splinting, those patients might be fast-tracked to surgery.
4. Regenerative and biologic therapies: Emerging treatments like nerve wraps, stem cell injections, or gene therapy that encourage nerve healing and regeneration represent a frontier for severe cases of CTS, especially those with nerve damage. Research into these modalities, including controlled clinical trials, could open new avenues for restoring nerve function without mechanical decompression.
5. Novel pharmacological approaches: There is an opportunity to explore medications that might prevent or reverse the pathologic changes in CTS. This could include drugs that reduce fibrosis in the carpal tunnel, improve microcirculation to the median nerve, or modulate inflammatory pathways more effectively and safely than current NSAIDs or steroids.
6. Collaboration and data sharing: Establishing multicenter registries or databases for CTS treatment outcomes would greatly enhance the power of research. By pooling data from numerous clinics and hospitals, researchers could more rapidly identify trends, rare complications, or effective nuances in technique, thereby accelerating improvements in care. Such collaborative studies could also facilitate the development of consensus guidelines and standardized outcome measures for CTS research.

In conclusion, carpal tunnel syndrome remains a highly prevalent condition that significantly affects patient quality of life, but the continuous evolution of its management offers hope for even better outcomes. By refining current treatments and exploring new therapies through rigorous research, the medical community aims to further reduce the impact of CTS and help patients maintain healthy hand function throughout their lives.

## Figures and Tables

**Table 1:** Comparison of CTS Surgery Techniques.

Technique	Incision Size	Advantages	Limitations
Open Carpal Tunnel Release	3–5 cm	Direct visualization; high success rate	Larger scar; longer recovery
Mini-Open Release	1.5–2 cm	Smaller incision; reduced tissue trauma	Less exposure; learning curve
Endoscopic Release	1 cm (1 or 2 portals)	Minimal scarring; faster recovery; less pain	Steeper learning curve; risk of incomplete release or nerve injury

**Table 2:** Comparison of CTS Conservative Management Modalities. (Data synthesized from peer-reviewed CTS studies. Refs. [13,14,15,16,17,18,19,20,21,22,23,24,25,26,27,28,29,30,31,32,33,34].)

Conservative Modality	Mechanism	Effectiveness
Wrist Splinting	Immobilizes wrist in neutral position to reduce median nerve pressure	Effective for mild cases; improves nocturnal symptoms
NSAIDs	Reduces pain and inflammation	Adjunctive symptom relief
Corticosteroid Injections	Reduces inflammation within carpal tunnel	Short-term relief (weeks/months)
Physical Therapy	Improves nerve mobility, strength, and ergonomics	Moderate short- to mid-term benefit; improves function

## Data Availability

No new data was generated.

## Abbreviations

The following abbreviations are used in this manuscript:

CTS	Carpal Tunnel Syndrome
NSAIDs	Nonsteroidal Anti-Inflammatory Drugs
PT	Physical Therapy
PRP	Platelet-Rich Plasma
MRI	Magnetic Resonance Imaging
